# A comparison of HAS & NICE guidelines for the economic evaluation of health technologies in the context of their respective national health care systems and cultural environments

**DOI:** 10.3402/jmahp.v3.24966

**Published:** 2015-03-12

**Authors:** Marc Massetti, Samuel Aballéa, Yann Videau, Cécile Rémuzat, Julie Roïz, Mondher Toumi

**Affiliations:** 1 Creativ-Ceutical, Paris, France; 2 ERUDITE - TEPP (Travail, Emploi et Politique Publique) FR CNRS 3126, Université Paris-Est Créteil Val-de-Marne, Paris, France; 3 Creativ-Ceutical, London, UK; 4 Public Health: Quality of Life and Chronic Diseases EA3279, Université de la Méditerranée, Marseille, France

**Keywords:** health technology assessment, guidelines, France, UK, economic evaluation

## Abstract

**Background:**

Health technology assessment (HTA) has been reinforced in France, notably with the introduction of economic evaluation in the pricing process for the most innovative and expensive treatments. Similarly to the National Institute for Clinical Excellence (NICE) in England, the National Authority for Health (HAS), which is responsible for economic evaluation of new health technologies in France, has published recommendations on the methods of economic evaluation. Since economic assessment represents a major element of HTA in England, exploring the differences between these methodological guidelines might help to comprehend both the shape and the role economic assessment is intended to have in the French health care system.

**Methods:**

Methodological guidelines for economic evaluation in France and England have been compared topic-by-topic in order to bring out key differences in the recommended methods for economic evaluation.

**Results:**

The analysis of both guidelines has revealed multiple similarities between France and England, although a number of differences were also noted regarding the elected methodology of analysis, the comparison of studies’ outcomes with cost-effectiveness thresholds, the study population to consider, the quality of life valuation methods, the perspective on costs, the types of resources considered and their valuation, the discount rates to apply in order to reflect the present value of interventions, etc. To account for these differences, modifications will be required in order to adapt economic models from one country to the other.

**Conclusions:**

Changes in HTA assessment methods occur in response to different challenges determined by the different philosophical and cultural considerations surrounding health and welfare as well as the political considerations regarding the role of public policies and the importance of their evaluation.

The health care spending of the developed countries has grown continuously in the course of the past few decades, particularly driven by the technological progress and medical innovation, the changes in behaviours of both patients and carers, as well as in clinical practice, but also boosted by the ageing of the populations which results in a higher prevalence of chronic disease and disability (1–3).

Many countries have implemented cost-containment measures, expressly targeting the pharmaceutical sector. In France, as soon as 1996, national targets for health expenditure (*Objectif national des dépenses de santé*, ONDAM) were implemented. In the United Kingdom, the National Institute for Clinical Excellence (NICE, now the National Institute for Health and Care Excellence) was set up in 1999 with the objective to reduce variation in the availability and quality of the treatments and care provided by the National Health Service (NHS) and started producing guidance taking into consideration issues of value for money. These initiatives stemmed in part from a willingness to emphasise the role of health technology assessment (HTA) and decision analysis in the pricing and reimbursement processes. Many HTA agencies have then integrated economic evaluation in the HTA process [the Norwegian Medicines Agency (NoMA), the National Centre for Pharmacoeconomics (NCPE) in Ireland, the Institute for Quality and Efficiency in Healthcare (IQWiG) in Germany, the Canadian Agency for Drugs and Technologies in Health (CADTH), the Belgian Health Care Knowledge Centre (KCE), the Scottish Medicines Consortium (SMC) in Scotland, the Dental and Pharmaceutical Benefits Agency (TLV) in Sweden, etc.] (4). More recently, the implementation of budgetary austerity policies in most of these countries has forced decision makers to favour efficiency in order to reduce the health care systems budgetary deficit.

In England, the NICE is mandated by the government to make recommendations on how to best allocate NHS budget. NICE provides multiple and single technology appraisals, taking into consideration clinical and economic data in order to inform the NHS on the opportunity represented by new health technologies. The NICE's technology appraisals alone determine the medico-economic value of health interventions. The main evaluation criterion for the economic evaluation of health interventions by NICE is the incremental cost-effectiveness ratio (ICER) expressed as incremental cost per quality adjusted life-year (QALY) gained with consideration to the established thresholds set at £20,000 and £30,000/QALYs (5).

In France, the National Authority for Health (Haute Autorité de Santé, HAS) is in charge of HTAs, performed by the Transparency Commission (*Commission de Transparence*, CT) for drugs, and the Medical Devices and Health Technologies Assessment Commission (*Commission Nationale d'Evaluation des Dispositifs Médicaux et des Technologies de Santé*, CNEDiMTS) for medical devices and other health interventions. These bodies evaluate the absolute and the added therapeutic value of health interventions in order to inform the decision-making agencies (economic committee for health products – *Comité Economique des Produits de Santé*, CEPS; National union of the medical insurances – *Union Nationale des Caisses d'Assurances Maladie*, UNCAM); Ministry of health). In 2012, the Economic Evaluation and Public Health Commission (*Commission d'Evaluation Economique et Santé Publique*, CEESP) was created within the HAS to conduct and review economic evaluations of health technologies and to inform the decision makers on the economic value and efficiency of the interventions assessed (6).

It has been debated whether or not France was introducing a NICE-like entity into its pricing and reimbursement process (7). To shed light on this matter, we reviewed in detail and compared the methods and processes for economic evaluation published by HAS and NICE. We identified similarities and differences between the recommended methods of evaluation regarding the assessment methodology, study population, comparators to include in the analysis, time horizon of the evaluation, measurement and collection of the data concerning health effects, costs perspective, resources considered and their valuation, discounting, and types of sensitivity analyses to perform. We then discussed the implications of those differences for adaptations of economic model between France and England.

These differences were then considered in the context of their respective national health care systems and political environment, revealing how the institutions and actors involved in HTA may have influenced the development of health economic evaluation guidelines. The aim of that part is to provide insight on the perception and influence of economic evaluation in these different environments.

## Guidelines comparison

We have compared the latest versions of the HAS and NICE methodological guidelines relative to economic assessment:

- Guide to the methods of technology appraisal. Published by NICE on April 2013 (8).
- *Choix méthodologiques pour l’évaluation économique à la HAS* (Choices in Methods for Economic Evaluation). Published by HAS in October 2011 (9).

The frameworks compared in the following article only concern NICE technology appraisals, thus excluding the more specific highly specialised technology (HST) evaluations that concern new and existing drugs for very rare conditions and interventional procedures (IP) for which specific guidelines have been published.

### General considerations

The two documents have similar structures. The topics addressed in the HAS guidelines match those in the NICE ones, which makes the comparison fairly straightforward. While NICE guidelines are descriptive, detailed, and prescriptive, the ones published by HAS are prominently non-exhaustive and non-definitive, leaving researchers with more flexibility to conduct economic evaluation. Both documents converge towards the same levels of requirements concerning data identification, production, and validation as well as methodological rigorousness. Both describe the methods that should be used to produce useful data in order to help in the decision-making processes for the committees in charge of making recommendations. Recommendations of both organisations favour the production of country-specific health outcomes, economic and quality of life data. HAS acknowledges the shortfall in local French data and recognises that time and budgetary constraints might lead to using foreign data and limit the feasibility of studies. Differences and similarities between the two documents are summarised in Table 1.

**Table 1:** Key similarities and divergences between the HAS and NICE methodological guidelines and processes for economic valuation of health interventions

HAS and NICE guidelines similarities	HAS and NICE guidelines divergences
CUA preferred analytical framework Subpopulations identification and analyses Comparators to take into consideration Time horizon Source of data for health effects of interventions Consideration of every positive and negative effect Effectiveness preferred over efficacy Sensitivity analyses	Possibility of a CEA (incremental cost per life-year gained) as base case in France Existence of cost-effectiveness thresholds Study population Quality of life valuation methods Perspective on costs Types of resources considered Valuation of the resources used Discounting Decision impacted by recommendation Place of the recommendation in the decision process

CUA: cost-utility analysis; CEA: cost-effectiveness assessment.

NICE produces TAs for interventions that are likely to have a significant health benefit, a significant impact on other health-related government policies, a significant impact on NHS resources, if significant variations in the use of the technology exist or if issuing national guidance is likely to add value. A positive appraisal will result in the inclusion of the assessed technology in the clinical commissioning groups’ formularies in order to be prescribed by practitioners to the patients. A negative recommendation will make a technology unlikely to be routinely funded by the NHS.

CEESP is in charge of assessing innovative health technologies that are likely to impact the expenses of the statutory public health insurance. This double condition limits the number of concerned interventions to the ones with significant added medical value (*Amélioration du service médical rendu*, ASMR) (10); that is, interventions for which a major, important or moderate level of ASMR (ASMR I–III) is claimed by the manufacturer in the reimbursement and pricing dossier submitted to the CT or CNEDiMTS, and which are expected to generate more than €20,000,000 annual sales revenue during the second full year of exploitation. Economic assessment is a new part of the pricing and reimbursement process in France and is still in its introductory stage; therefore, it may not be extended to cover all health technologies in a short timeframe.

Both HTA agencies produce information on the costs and effectiveness of new health interventions. However, their recommendations lead to different decisions and do not have the same leverage in the decision process.

NICE technology appraisals determine whether the use of the assessed technologies should be recommended for the NHS in England. In case of a positive recommendation, the technology becomes available in the national formulary and clinical commissioning groups are then legally obliged, within 3 months, to provide it to the patients.

In France, the cost-effectiveness of the concerned interventions is reported to the economic committee for medicinal products (CEPS) in the form of an ‘efficiency report’ (*Rapport d'efficience*). The efficiency is then considered alongside the relative therapeutic value and other considerations (industrial concerns, support of innovation and research, public health requirements, etc.) during the pricing negotiations held between the CEPS and the manufacturer.

### Authoring teams

A variety of actors of the health care system have been involved in the process of writing the guidelines, including medical practitioners, pharmacists, epidemiologists, economists, and public health specialists, working in both public administrations and private sectors. However, the organisation of the authoring teams varies slightly between the documents: NICE's guidelines were supervised by NICE staff members, mostly with a background in pharmacy and a steering group involving a mix of medical doctors, health economists, pharmacists and economists, lay representatives, and manufacturer representatives. The authoring team of the HAS’ guidance was supervised by economists and included a mix of medical doctors with an expertise in economics and public health, statisticians, pharmacists, and economists, as well.

### Type of economic evaluation

In order to maximise health gains under the health care systems’ budget constraints, NICE recommends the use of cost-utility analyses (CUA) for all interventions, whereas the HAS recommends CUA only for interventions with a significant impact on health-related quality of life (HRQoL). If HRQoL is not identified as a relevant health effect of the studied interventions, then cost-effectiveness analysis (CEA) is the required economic evaluation. CEA is also accepted as base-case analysis in France if HRQoL data are not available or cannot be produced at reasonable cost within reasonable time. The HAS also acknowledges that the QALY approach might not be the best suited for specific fields, in which case, cost-effectiveness studies might be more appropriate. Both agencies have rejected the use of cost-benefit analyses for ethical concerns, and agree that QALYs must be valued from the general population perspective.

In England, economic evaluation of health technologies needs to provide outcomes expressed as incremental costs and QALYs generated by the assessed treatment strategies. ICERs of the assessed interventions are compared to cost-effectiveness thresholds. NICE rejects the concept of an absolute threshold for judging the level of acceptability of a technology in the NHS, as there is no practical basis for deciding at what value a threshold should be set. In some cases, NICE may wish to take into consideration other factors than the defined thresholds. Interventions with ICERs below £20,000/QALY are considered cost-effective for the NHS. Those with ICERs between £20,000 and £30,000/QALY must provide additional data in their favour in order to be recommended for use in the NHS. Interventions with ICERs above £30,000/QALY are required to make a stronger case in order to gain positive recommendation by NICE.

In France, the preferred health outcome in situations where CEA is best suited is the survival rate, expressed in calculated life-years and accounting for all causes of mortality. If the survival rate is proven to be unsuitable or impossible to determine, the final clinical outcomes such as complication rates should be used. If not possible, the resort to survival-related surrogate outcomes must be well justified and the used outcomes must be validated. However, such situations will likely be rare, in view of the fact that the interventions eligible for economic assessment claim a high level of ASMR. No established threshold in terms of incremental cost per QALY or per life-year gained is employed. However, results of the sensitivity analyses assessing the uncertainty around the costs and effects of the intervention can be presented as acceptability curves.

### Study population

The population considered in NICE's appraisals is the population specified in the marketing authorisation. Besides the health effects on the patients, the impact of the treatment on their caregivers can be taken into account in the appraisal, if appropriate. In France, HAS requires the technology assessments to consider all individuals whose health is directly or indirectly affected by the intervention studied (e.g., non-vaccinated population in case of vaccines, possible development of resistance affecting future patients in case of antibiotherapy).

In both countries, subgroups of interest might be highlighted among which clinical or cost-effectiveness of the technology is expected to differ from the general population or if some subpopulations require specific considerations.

### Comparators

All potentially relevant interventions for the assessed indication should be compared, whether they have a marketing authorisation in the concerned indication or not. HAS recommends comparing the new product to current best practice and consensus/routine treatment. It also considers as useful the comparison to the emerging practices, the best supportive care, and the no intervention option, as such analyses may reveal lower efficacy of the current standard of care. All potentially relevant comparators are determined by the NICE during the scoping process at the beginning of each appraisal. Appropriate comparators are defined as established practice in the NHS, taking into account the natural history of the condition without treatment, existing NICE guidance, cost-effectiveness, and the licensing status of the comparator.

### Time horizon

For both agencies, the time horizon of the economic assessment should be long enough to capture the whole impact that interventions have on health of the concerned population as well as their associated costs. A lifetime horizon is recommended by both agencies when the assessed technologies have an impact on survival or have lifelong consequences in terms of costs or health outcomes. Shorter time horizons can be justified in cases of acute diseases without long-term sequels. The HAS guidelines also mention the possibility of multigenerational time horizons for specific interventions such as vaccines.

### Health effects

In order to determine the impact of an intervention on patients’ health, a systematic literature review should be performed to comparatively describe its effects and all the relevant studies available should be considered. Both agencies favour head-to-head randomised controlled trials (RCT) which directly compare the studied interventions. Data from non-randomised studies may add value to the results, limit bias, or provide some additional information. Expert opinions cannot be used directly to measure health effects, although they might be used to document other dimensions of the assessment when no data of satisfactory quality can be retrieved or contextualise the effects observed. The generated evidence on health effects is synthesised in a meta-analysis. Results should be selected and critiqued, and heterogeneity should be assessed. Where no head-to-head RCT exists, mixed treatment comparison using network meta-analysis of RCTs can be performed. In case national data are unavailable, issues of transferability and consistency of foreign data with the local setting should be discussed.

All the effects of the assessed interventions on health must be identified and taken into consideration in the economic assessment, whether they are positive effects or negative. Every effect should then be valued adequately in terms of QALYs, or alternative outcomes such as life-years in France. Economic evaluations should reflect the effect of the treatment in real life (effectiveness) rather than in the particular conditions of a clinical trial (efficacy). Since effectiveness cannot usually be measured in RCTs, effectiveness should be derived from efficacy through modelling. Similarly, the biases limiting transferability and generalisability of the results should be discussed and addressed if possible.

### Valuation of health outcomes

For NICE, EQ-5D is the preferred measure for HRQoL in adults, while HAS will exclusively consider HRQoL data generated from generic instruments which are validated in France and for which sets of preference weights elicited from the French general population are available (for now, HUI-3 and EQ-5D). In both countries, HRQoL associated to health states should be measured in patients, while preferences should be elicited from a representative sample of the general population. NICE authorises mapping other HRQoL measures to EQ-5D when no EQ-5D data are available, provided that it is justified, that mapping functions are validated and that sensitivity analyses are performed, while HAS notes that no existing mapping function was shown to be valid in France and could therefore be used in the base case analysis.

In France, the HAS acknowledges the lack of French utility data. If such data cannot be found or produced, foreign data based on EQ-5D or HUI-3 with good methodological quality may be used, although the transferability of such data should be discussed. HRQoL descriptions from foreign patients can also be re-weighted using preference weights elicited from the French general population. When the generic instruments lack sensitivity in the situation analysed, supplemental data obtained with more appropriate HRQoL questionnaires may be provided.

### Costs

NICE and HAS’ respective views on costs differ: in England, costs should be considered from the sole perspective of the National Health Service and Personal Social Services (NHS & PSS) perspective whereas HAS desires to embrace a more collective ‘all payers’ perspective, taking account of every stakeholders concerned by the decision of the fund supplier for the health care system.

This leads to differences in the resource utilisation considered (Table 2). In England, only resources under the control of the NHS and PSS should be included in the base-case analysis and valued relevantly to the NHS and PSS. Costs borne by patients and reimbursed by the NHS and PSS may be included in the base case analysis as well. Health care costs due to extra survival rates should be included when they result of the indication treated by the assessed interventions. Costs due to investment or infrastructure modifications should be included. Health care resources that are not funded by the NHS and PSS should not be included in the base case analysis; nevertheless, they can be added in a supplementary analysis. Productivity costs should be excluded from the study. Carer's time should not be valued and included in the base case analysis. However, when carer's implication might have been provided by NHS and PSS, the corresponding time can be valued relevantly and the resulting costs included in a separate analysis. In France, the base case analysis is limited to the direct costs, that is, costs related to the resources used in the production of the intervention: consumption of hospital care, outpatient care, medical goods, transport, organisation of a health care program, time spent by people undergoing the interventions, and time spent by their care givers, as well as costs related to the treated disease during the added life-years. Transition costs must also be presented (resources consumption required for the intervention to be routinely used, including infrastructure modifications). The indirect costs may be analysed separately. They include resources and time used because of mortality/morbidity, measured as the duration of the different categories of activities affected.

**Table 2:** Costs considered in the base case economic analyses as recommended by NICE and HAS guidelines

Costs	HAS	NICE
Inpatient care	✓	✓
Outpatient care	✓	✓
Drugs and other medical goods	✓	✓
Emergency and specific patient transport	✓	✓
All costs linked to travel	✓	
Care for elderly persons in institution	✓	✓
Organisation of a public health program	✓	✓
Disability compensation program	✓	✓
Future costs related to the treated disease	✓	✓
Costs due to investment and infrastructure modifications	✓	✓
Other transition costs borne by other health care system stakeholders	✓	
Patients’ and carers’ time dedicated to the intervention	✓	

To sum up, the key differences between cost analyses performed in France and in England would be that:

- Costs paid by patients or private insurance are considered in France only.
- Time spent to receive health care interventions is included in France.
- Time spent by carers is more widely considered in France than in England.

In the United Kingdom, evaluation of resource use is based on a public list of prices for the technologies, taking into consideration nationally available price reductions and patient access schemes (PASs). Prices of medicines prescribed in primary care should be based on Drug Tariffs. Inpatient care costs can be based on DRG, microcosting, or literature reviews, where appropriate. Because of the HAS choice of the ‘all payers’ perspective, resource utilisation should be estimated using production costs. If unavailable, tariffs may be used, including expenditures over and above the tariffs and documenting the potential difference between tariffs and production costs. The HAS wishes to be in a position to identify the distribution of the expenditure between payers as well as to detect the potential changes that the introduction of a new technology might bring about. When it may represent a significant cost, the time cost for the carers and the patients should also be estimated.

### Discounting

Costs and health effects should be discounted at an annual rate of 3.5% in England and 4% in France to reflect their present value. Both organisations require performing sensitivity analyses using alternative discount rates (1.5% in England, 3 and 6% in France). In France, after 30 years, the discount rate should be progressively decreased to 2% while NICE allows for lower rates to be applied in the case of therapies with long-term health benefits (i.e., 1.5%).

### Sensitivity analyses

Both HAS and NICE require sensitivity analyses in order to assess uncertainty around the costs and effectiveness estimates. In both countries, probabilistic sensitivity analyses (PSA) are preferred. If the model structure and/or way of implementation limit the feasibility of PSA, this should be specified and justified. However, the choice of a model structure and programming should not result in a failure to determine uncertainty. PSA results can be reported in the forms of confidence ellipses and scatter plots on the cost-effectiveness plane, acceptability curves, and detailed tabulated results. Correlation/independence between individual parameters should be discussed. In England, univariate and multivariate deterministic sensitivity analyses (DSA) can be performed in order to produce knowledge on the main sources of variability and orient further outcomes research. DSA are mandatory in France (8, 9).

## United Kingdom–France model adaptations

The scopes of interventions concerned by economic assessment in France and England are not similar, so it is expected that interventions requiring assessment in one country will not systematically need to provide economic data in the other. However, most interventions that require an economic evaluation by HAS will likely be evaluated in England, except for some interventions excluded from the remit of NICE (such as vaccines, which are evaluated by the Joint Committee on Vaccination and Immunisation).

Beyond the need to produce nationally relevant results, there may be different comparators or treatment pathway requirements in one country, or different epidemiological situations, calling for changes in the model structure. But are there differences in the methods of economic evaluations that would lead to changes in model structures and in results? The differences identified throughout the guidelines of both agencies in terms of methodology and scope of the analysis may hinder the transferability and adaptation of models and results from one country to another.

One major divergence between the methodological recommendations of NICE and HAS concerns the analytical framework: while NICE wishes to assess all interventions on the basis of cost-utility analysis results, HAS recognises that CEA might be more appropriate in a number of situations, and particularly when the intervention has an impact on survival but not on quality of life. This might result in structural modifications being brought to the models in various cases, notably in the field of oncology or other end-of-life treatments for which HRQoL might not be appropriate or representative of the impact that health interventions would have on patient's health. In reality, most interventions will have some impact on quality of life (whether related to symptom improvement or adverse events) even if the main objective of treatment is to improve survival. Therefore, when the main benefit of a product is to improve survival, manufacturers will have some flexibility to choose between presenting to HAS an incremental cost per life-year gained or an incremental cost per QALY gained; the former ratio may be lower in many situations as it does not incorporate health impairment during the added years of life.

Study population is also subject to methodological differences in terms of the scope of people concerned by the interventions’ effects and costs. Models developed for HAS might require additional analysis and data gathering in this regard, taking into consideration all individuals whose health is directly or indirectly affected by the intervention. This includes carers (although NICE too wishes the carers to be included in the study population when their health is affected by the intervention), but HAS cites also the case of populations indirectly concerned by costs and effects of interventions, such as non-vaccinated populations, or populations negatively affected by the use of antibiotherapy. Although vaccines and vaccination campaigns are out of NICE assessment scope, this illustrates the more comprehensive framework that might be required by HAS in the modelling process. The consideration of populations negatively affected by the use of anti-infectious therapy might also reflect a specific concern for this public health issue from the French authorities due to the high national consumption of antibiotics in both inpatient and outpatient settings (11).

Differences concerning the perspective on costs and the differences in terms of resources consumption considered in the analyses will require structural changes between the models, notably in order to identify the different payers involved in the health care system, to determine the modifications in the cost-distribution between the different stakeholders and take into account the time costs for receiving health care and time of carers. This could have major impact on the cost of some interventions, particularly, in the comparison of interventions requiring hospital admission and interventions received at home. It also raises many questions not specifically addressed in the HAS guidelines, such as whether the time costs related to health care for disease avoided should be considered; or whether time spent to receive health care during illness periods should be considered and evaluated in the same way as for health care received during healthy periods. Whereas the first question should be considered in the HAS guidelines, from our interpretation, the latter might be considered as double counting, since ill time is also estimated in terms of QALYs lost. In addition, the HAS does not provide any recommendation on how and on what basis time should be estimated, and merely notes that this is a difficult task, with limited data sources. Estimating time on the basis of lost productivity is probably too restrictive, and could lead to inaccurate estimates of the interventions’ value for working individuals.

Overall, it appears that models developed for HAS might differ slightly from the models designed for the NICE by requiring additional considerations around costs and expanding the study population (assuming treatment pathways and epidemiological situation are similar between countries). However, the differences identified relate mostly to parameters’ estimation rather than structural modifications and should be easily amended.

## Impact of economic evaluations

The role of economic evaluation in HTA and within the decision process differs greatly between France and England. In the latter, economic evaluations appear to have a major influence in the evaluation and decision process (that can be perceived as merged). In a research paper, the ICER alone was found to correctly predict 82% of NICE decisions (12). The question NICE is mandated to answer is ‘should a technology be employed within the established limited NHS budget?’ NICE does not statute as whether additional resources should be made available in order to fund the introduction of an intervention. The government can question an expensive intervention even though NICE has assessed it as cost-effective. The question of the opportunity cost of the intervention is asked in the context of the sole NHS budget.

On the contrary, in France, health economic evaluations are considered in the context of the pricing negotiations held between the manufacturer and the CEPS. Efficiency notices are designed as supplementary data involved in the decision-making alongside the budget impact of the technology, its additional therapeutic value, or other criteria such as industrial matters and support to innovation (13). More efficient technology may claim a higher price given the consequences it may have on the various health care payers (as these are explicitly detailed in the assessment). The choice as to whether or not a new intervention should be supported by the health care system relies on the UNCAM, which uses advice on the absolute and additional therapeutic value of the product by the CT/CNEDiMTS (14).

NICE evaluations assess the efficiency of the interventions studied and their uncertainty in quantitative terms, while HAS does not rule on the efficiency of new interventions or the acceptability of their opportunity cost in quantitative terms in the efficiency notices: the assessments performed by the CEESP address the conformity of the analyses submitted by the manufacturer relatively to the methodological requirements and recommendations, a proxy of the uncertainty surrounding the relevance of the submitted results in the context of the French health care system. The CEPS has the responsibility to conclude on the efficiency of the intervention in regard of the results produced in the submitted efficiency dossier and their evaluation by the CEESP.

However, the framework agreement between the CEPS and the pharmaceutical industry has planned to base the pricing negotiations on external reference pricing for new drugs considered as of moderate to high added therapeutic value (ASMR levels I–III, i.e., drugs concerned by economic evaluation) (13). Therefore, efficiency notices and economic assessments might find a limited practical utilisation in the near future, as a lever in the negotiation of rebates consented by the manufacturer on the retail price, similarly to what has been introduced in 2009 in the NHS with PASs. PASs consist in rebates agreements between the Department of Health and a company applying for recommendation in the NHS in order to improve a drug's cost-effectiveness. Their negotiation involves input from the dedicated Patient Access Scheme Liaison Unit (PASLU) among NICE and are susceptible to allow positive recommendations from NICE. Several types of PASs have been set up in England: simple discounts on product's turnover or free stock supply to NHS, rebates based on dose caps, on patients’ response towards treatment. In France, rebates are one of the instruments that the CEPS uses in order to contain the growth of the pharmaceutical expenditure as targeted by the legislative authority. They consist of price revision clauses as well as products and class sales volumes agreements that lead to the reimbursement of rebates by the manufacturer when the forecasted sales volumes are exceeded. Discounts on the products public price are also negotiated between the manufacturer and the CEPS.

Since the CEESP evaluations communicated to the CEPS do not report any quantitative results (acceptable price considering the indication, treatment population, public health need, or particular costs for the French sick fund), it still remains unclear how economic assessment will be used in the rebates negotiation process with the manufacturer. Eventually, experience should lead to more pragmatism, notably with a more specific definition of the importance and role of economic evaluation in the framework agreement between the CEESP and the pharmaceutical industry.

## Conclusion

Differences in the basic principles underlying the health care systems, the structure of medical practice, as well as the philosophical and cultural notions of health, disease, and medicine exist between France and England. Those may affect the role economic evaluation is set to take in the recommendation and decision process of these countries (15).

In England, the Beveridgian National Health Service was designed as a provider of universal access to health care. This service has an allocated budget financed by every citizen through taxation. While in France, the health care system is part of a wider Bismarckian social security service that aims to guarantee individual right of access to the same level of health care and innovation to all through contribution and redistribution. The French statutory health insurance is a third party payer, whose expenses are framed by spending targets (15). The NHS being framed by a defined allocated budget, rationing became necessary and economic evaluation-based HTA was accepted as a way to rationalise rationing in a system where efficiency of provided care is a major concern for their suppliers and recipients (16). However, in a context where French social security and its basic principles remain at the core of the French social pact and political decisions, health economics-based HTA and assessment of efficiency can be perceived as a way to introduce rationing in a system that is not intended to limit access to the most effective care for its beneficiaries (15, 16). In particular, the definition of an ‘efficiency-threshold’ above which patients would see the newer – and sometimes better – treatments not reimbursed to them would not be compatible with the definition and original principles of the French social security. Thus, the limitation of the scope of the economic assessment of health technologies to the pricing negotiations appears coherent, in a context where reimbursement of the most effective health technologies cannot be conditioned on economic criteria. As another consequence, the sole public statutory health insurance perspective cannot be perceived as appropriate in France: since the health care system is supposed to guarantee equal access to care to every citizen, the expenses that are reimbursed by the private complementary insurances and the patients’ out-of-pocket expense must be considered in the analysis as well.

Health care in England is mainly organised around the general practitioners (GPs) who are contracted by the NHS and paid by capitation alongside pay for performance measures favouring their agreement to good practices scheme established by NICE. The GPs act as residual claimants towards efficiency: they are perceived and perceive themselves as care managers, reinforcing their commitment towards efficiency. On the opposite, in France, the medical practice is more disaggregated between specialists and GPs. Most GPs practice medicine on a self-employed status and are remunerated on a fee-for-service basis that does not favour efficiency, which remains centralised at the level of the health authorities and hardly succeeds to become a local concern for practitioners (17, 18). Furthermore, a high level of standardisation of care has been developed in England, allowing the reduction of uncertainty surrounding medical practice, thus giving more strength and impact to economic evaluation and increasing its potential added value (4), while French medical practitioners are bound to their freedom of practice as claimed in 1927 by the Confederation of French medical Trade Unions, and they do not feel comfortable with the standardisation of care, notably because of ethical concerns (19). Thus, variability exists between their practices, which may reduce the significance and credibility of health economic studies, limiting the importance and role regarding the decision process.

The differences in the philosophical and cultural concepts of health, disease, and medical practice that support the bases of the national health care systems can also be considered to explain the different frameworks of economic evaluation in health care between the two countries. In England, logical positivism as developed originally by Claude Bernard on the bases of Auguste Comte's work can be perceived as the dominant philosophy regarding health. Such an approach implies that health is a deterministic concept that only differs from illness on a quantitative point of view (15). As a consequence, health and disease can be represented as quantitative parameters in the context of a study. These notions allow for a better consideration of standardisation of care and of the premise that, if enough parameters are accounted for, the consequences of the introduction of a new health intervention can be forecasted accurately enough to take part in the assessment and decision process. In France, George Canguilhelm's considerations around health and disease substituted to those developed by the positivists. Behind his theory lies the notion that every patient is singular in his disease and the way he experiences it and must therefore be taken care of independently (20). Canguilhelm's ideas being dominant in the medical and political elites of France, standardisation of care and mathematical representation of diseases are criticised, limiting the scope and impact of health economic evaluations among the practitioners and decision makers (15). This opposition in the ideas of health and illness might also account for a contrasted perception of health-related utilitarianism between the elites and decision makers of the two countries. In England, logical positivism might have acted in its favour and a certain teleological moral advocating for the determination of intervention maximising the well-being, using tools such as CEAs, valuing welfare based on quality of life (utility), or monetary values. These considerations are largely contradicted by the ones of Canguilhelm and a Kantian, deontological (as opposed to teleological) morality, more widespread in France, according to which, what is good and constitutes welfare cannot be defined, thus forbidding making value judgments regarding individuals’ life conditions (21). This situation may lead to an increased criticism towards QALYs on ethical and technical bases in France, thus hindering the development of QALYs and their consideration by the medical and political elites (22).

Despite the gap between the French and English health care systems’ bases and organisation, HAS and NICE methodological guidelines are relatively similar, supporting the idea that harmonisation of recommendations concerning economic evaluation in Europe is possible. The convergence between countries already appears to be happening, for example with the changes in the role and modality of exercise of GPs and the introduction of economic evaluation in France or the development of PASs, introducing price negotiation in England, as well as the inclusion of disease burden and wider societal considerations in the appraisal process.

The culture of evaluation in the field of public policies is still in its early development in France, and the consideration of economic assessment in the decision-making process, notably in the field of health care, is very recent, increasing the need for further research analysing the impact of economic assessment on the decisions taken and on the prescribers’ behaviour as well.
